# Development & Testing of a Comprehensive Digital Self-Care Support System for Older Adults With Chronic Conditions

**DOI:** 10.1093/geroni/igab046.2398

**Published:** 2021-12-17

**Authors:** Priya Nambisan, Kurt Stange, Kalle Lyytinen, Eva Kahana, Edmund Duthie, Michael Potnek

**Affiliations:** 1 University of Wisconsin- Milwaukee, Milwaukee, Wisconsin, United States; 2 Case Western Reserve University, Cleveland, Ohio, United States; 3 Medical College of Wisconsin, Milwaukee, Wisconsin, United States; 4 Outreach Community Health Center, Milwaukee, Wisconsin, United States

## Abstract

This 3-phase study involves the conceptualization and design, development and usability testing of a Comprehensive Digital Self-care Support System (CDSSS) named myHESTIA for older adults with multiple chronic conditions (MCC). The objective of this study was to test whether a CDSSS can be developed for those who are dealing with MCC and whether such a system that is specifically developed for older adult patients will enable daily capture of self-care data. Participants for this 3-phase study included: 10 older adults (age>60) and 10 caregivers in Phase 1; 15 Geriatrics clinicians and 25 community-dwelling low-income older adults in Phase 2; and, 10 older adults (age>60) with MCC in Phase 3. Agile method of system development was used for the design and development of the system. The first two phases involved collecting data for designing and developing myHESTIA. The third phase involved small group usability and feasibility testing, in which the participants used myHESTIA trackers for 4 weeks. Results from phase 3 shows daily inputs were possible and the self-reported data shows that it was not at all difficult for older adults to track their symptoms daily. User experience data (n=10) shows overall positive experience along pragmatic (5.8 out of 7), hedonic (4.6 out of 7), sociability (5.5 out of 7) and usability (6.3 out of 7) experience dimensions. Finally, all the participants (n=10) who completed the phase 3 study reported intention to continue using myHESTIA. Results indicate that it is feasible to design a CDSSS for older adults with MCC.

